# Glycation in Alzheimer’s Disease and Type 2 Diabetes: The Prospect of Dual Drug Approaches for Therapeutic Interventions

**DOI:** 10.1007/s12035-025-05051-9

**Published:** 2025-05-22

**Authors:** Sama Ayoub, Maryam Arabi, Yousef Al-Najjar, Ibrahim Laswi, Tiago F. Outeiro, Ali Chaari

**Affiliations:** 1 Weill Cornell Medicine–Qatar, Qatar Foundation, Education City, P.O. Box 24144, Doha Qatar; 2 Department of Internal Medicine, Yale New Haven Hospital, New Haven, CT USA; 3 Department of Experimental Neurodegeneration, Center for Biostructural Imaging of Neurodegeneration, University Medical Center Göttingen, Göttingen, Germany; 4 Translational and Clinical Research Institute, Faculty of Medical Sciences, Newcastle University, Framlington Place, Newcastle Upon Tyne, Newcastle, NE2 4HH UK; 5 Max Planck Institute for Multidisciplinary Sciences, Göttingen, Germany; 6 Scientific Employee With an Honorary Contract at Deutsches Zentrum für Neurodegenerative Erkrankungen (DZNE), Von-Siebold-Straße 3a, 37075 Göttingen, Germany

**Keywords:** Alzheimer’s disease, Type 2 diabetes, Advanced glycation end products (AGEs), Dual drugs

## Abstract

As global life expectancy increases, the prevalence of neurodegenerative diseases like Alzheimer’s disease (AD) continues to rise. Since therapeutic options are minimal, a deeper understanding of the pathophysiology is essential for improved diagnosis and treatments. AD is marked by the aggregation of Aβ proteins, tau hyperphosphorylation, and progressive neuronal loss, though its precise origins remain poorly understood. Meanwhile, type 2 diabetes mellitus (T2DM) is characterized by chronic hyperglycemia, leading to the formation of advanced glycation end products (AGEs), which are implicated in tissue damage and neurotoxicity. These AGEs can be resistant to proteolysis and, therefore, accumulate, exacerbating AD pathology and accelerating neurodegeneration. Insulin resistance, a hallmark of T2DM, further complicates AD pathogenesis by promoting tau hyperphosphorylation and Aβ plaque accumulation. Additionally, gut microbiome dysbiosis in T2DM fosters AGE accumulation and neuroinflammation, underscoring the intricate relationship between metabolic disorders, gut health, and neurodegenerative processes. This complex interplay presents both a challenge and a potential avenue for therapeutic intervention. Emerging evidence suggests that antidiabetic medications may offer cognitive benefits in AD, as well as in other neurodegenerative conditions, pointing to a shared pathophysiology. Thus, we posit that targeting AGEs, insulin signaling, and gut microbiota dynamics presents promising opportunities for innovative treatment approaches in AD and T2DM.

## Background

With global life expectancy now surpassing the eighth decade, the prevalence of age-related neurodegenerative diseases is expected to increase dramatically [1]. Neurodegenerative disorders, which impair the normal functioning of the nervous system, manifest in a variety of debilitating symptoms, including loss of voluntary movement, coordination, cognition, sensation, or strength.

By 2050, an estimated 152 million people will be living with AD and other forms of dementia [2]. Pathologically, AD is characterized by the extracellular accumulation of Aβ plaques, intracellular tangles of hyperphosphorylated tau protein, and widespread neuronal loss leading to brain atrophy. Co-pathologies, including alpha-synuclein and TDP-43 protein deposits, are also evident in AD cases [3]. However, despite decades of research, the precise mechanisms underlying AD pathogenesis remain elusive [4].

Current pharmacological treatments provide symptomatic relief but fall short of a cure, underscoring the urgent need for early diagnosis and more effective intervention strategies. Similarly, T2DM has reached epidemic proportions, with over 400 million individuals affected globally [5]. This chronic metabolic disorder is marked by insulin resistance, impaired insulin secretion, and hyperglycemia [6]. T2DM is associated with a range of complications, including cardiovascular disease, nerve damage, kidney failure, and vision impairment, and it is often accompanied by central obesity, hypertension, and dyslipidemia—features collectively referred to as metabolic syndrome. The widespread prevalence of T2DM presents significant challenges to public health, not only due to its complications but also because of the profound economic burdens linked to healthcare costs and productivity loss.

The complex relationship between AD and T2DM has received growing interest recently. Emerging evidence from clinical and epidemiological studies suggests that individuals with metabolic disorders such as T2DM, obesity, and hypertension are at an increased risk of developing neurodegenerative diseases like AD [7]. This intricate relationship is underpinned by shared risk factors, overlapping pathophysiological mechanisms, and potential therapeutic parallels. Therefore, understanding these connections is crucial for devising effective prevention and treatment strategies that may impact both conditions.

One critical pathological link between AD and T2DM is the process of protein glycation, in which sugars irreversibly modify proteins, lipids, or nucleic acids, forming advanced glycation end products (AGEs) [8]. These AGEs play a critical role in the progression of both diseases. In T2DM, chronic hyperglycemia accelerates glycation, accumulating AGE, contributing to insulin resistance, vascular damage, and tissue injury. Similarly, in AD, AGEs accumulate in the brain, exacerbating amyloid plaque formation, promoting oxidative stress, and inflicting neuronal damage. Given the central role of AGEs in both conditions, targeting glycation pathways can significantly mitigate disease progression and improve global health outcomes.

This review examines the shared pathological roles of AGEs in AD and T2DM, highlighting potential therapeutic overlaps. We explore the glycation process and its contribution to Aβ toxicity and cognitive decline in AD. We also investigate the influence of detoxifying enzymes and gut microbiota on AGE levels and neuroinflammation in both diseases. Additionally, we discuss the therapeutic potential of anti-diabetic medications in the context of AD, as these drugs, which are primarily used to manage hyperglycemia, have shown promise in targeting shared molecular pathways between the two conditions. In total, we aim to identify new clinical strategies that leverage the interconnected nature of diabetic and neurodegenerative diseases, possibly by affecting neuropathology, to improve patient outcomes.

## AD Neuropathology

AD is characterized by a combination of positive and negative neuropathological features alongside chronic neuroinflammation, which collectively contribute to the progression of the disease. The positive lesions, including amyloid-beta (Aβ) plaques and neurofibrillary tangles (NFTs) composed of hyperphosphorylated tau, disrupt neuronal communication and intracellular transport. Negative lesions, such as synaptic loss and neuronal death, result in widespread brain atrophy and cognitive decline. Additionally, neuroinflammation driven by the activation of microglia and astrocytes and the release of pro-inflammatory cytokines intensifies neuronal damage. Together, these processes highlight the complexity of AD, where multiple cellular and molecular mechanisms converge to drive neurodegeneration. Understanding these interactions is critical for developing targeted therapies to slow disease progression and improve the quality of life for affected individuals.

### Positive Lesions

Positive lesions in AD are defined by the presence of Aβ deposits and NFTs, both of which play pivotal roles in the disease’s progression. Aβ peptides, generated through the cleavage of amyloid precursor protein (APP) by β-secretase and γ-secretase, aggregate to form plaques. Among these peptides, Aβ42 is particularly neurotoxic due to its higher propensity for fibrilization, and decreased solubility compared to Aβ40, which is more abundant but less harmful [9, 10]. The accumulation of these plaques leads to neuronal damage and cognitive decline.

NFTs, composed of hyperphosphorylated tau, also contribute to AD pathology. The hyperphosphorylation of tau disrupts its interaction with microtubules, destabilizing the cytoskeleton and impairing synaptic communication. This disruption, driven by kinase dysregulation, oxidative stress, and inflammation, correlates closely with dementia severity [11, 12]. Together, Aβ plaques and NFTs represent critical therapeutic targets for AD.

### Negative Lesions

Neuronal and synaptic loss occurs early in AD, before the appearance of amyloid plaques and NFTs. This loss exceeds the rate of normal aging and contributes significantly to brain atrophy and cognitive impairment. Synaptic degradation and neuronal death are linked to impaired neurogenesis, especially in regions like the hippocampus, which exacerbates cognitive deficits [13, 14].

Further, the loss of allopregnanolone, a neurosteroid, has been shown to worsen frontal cortex dysfunction, contributing to impaired cognition [15]. The increased apoptosis rate in AD distinguishes it from the natural aging process, highlighting the urgency of early intervention to slow neuronal and synaptic loss [16]. Identifying these early negative lesions can help in developing preventative strategies for AD-related neurodegeneration.

### Neuroinflammation

The accumulation of Aβ plaques and NFTs in AD initiates a cascade of neuroinflammatory responses. Microglia and astrocytes become activated, releasing pro-inflammatory cytokines that, in turn, contribute to neuronal damage. While some of these cytokines may have protective effects under normal circumstances, their excessive production exacerbates neurodegeneration. Vital inflammatory pathways, including nitric oxide, cyclooxygenase (COX), and caspase signaling, amplify cytokine release, further promoting the inflammatory cycle [17, 18].

In addition to the direct effects of glial activation, dysbiosis in the gut microbiome has been linked to neuroinflammation in AD. Imbalances in gut microbiota can produce harmful molecules like lipopolysaccharides (LPS) and amyloids, which penetrate the blood–brain barrier, activating the NFκB signaling pathway and accelerating neuroinflammation [19]. Furthermore, chronic hyperglycemia in T2DM promotes excessive formation of advanced glycation end-products (AGEs), which accumulate in the brain and bind to the receptor for AGEs (RAGE) on neurons, microglia, and endothelium [20]. RAGE engagement potently activates NF-κB and NADPH oxidase signaling, greatly amplifying oxidative stress (via ROS) and the release of pro-inflammatory cytokines (e.g., TNF-α, IL-6) [21]. This injurious oxidative–inflammatory milieu impairs synaptic function and drives neuronal apoptosis [22]. In parallel, hyperglycemia and RAGE-dependent signaling activate PKC/NF-κB cascades that disrupt tight junctions and blood–brain barrier integrity [23] permitting influx of peripheral immune factors and Aβ into the CNS. Together, these interlinked mechanisms directly link diabetic hyperglycemia to exacerbated AD neuroinflammation and accelerated neuron loss. This complex interplay between systemic inflammation, gut microbiota dysbiosis, and neurodegeneration underpins the multifaceted nature of AD and offers promising avenues for therapeutic intervention. Although targeting AGEs and RAGE shows some promise, it is unlikely to be sufficient alone to significantly alter Alzheimer’s disease AD progression. Recent studies support multi-targeted approaches, combining anti-glycation strategies with therapies against other pathological mechanisms, to achieve better cognitive outcomes and broader disease modification. While these strategies introduce greater complexity, they align with the multifactorial nature of AD, and current research increasingly favors integrated interventions over single-target therapies.

## Neuropathology and Protein Glycation in AD

AD has been closely linked to the accumulation of AGEs in the brain. This association has fueled growing interest in developing therapies aimed at mitigating AGE-induced neurodegeneration. Despite ongoing research, no effective treatments for AD have yet emerged. Understanding the intricate relationship between AGEs and AD pathology is critical for devising effective therapeutic strategies against the disease.

A promising area of research focuses on specific AGEs, such as carboxymethyl lysine (CML) and carboxyethyl lysine (CEL), which are elevated in the serum of individuals with AD. These AGEs are produced both endogenously and through dietary intake. Preclinical studies suggest that reducing dietary AGEs may influence AD pathology, particularly in older adults with T2DM. However, data on the direct correlation between serum and brain levels of AGEs and AD pathology in humans remains limited.

Lifestyle interventions, particularly diet and physical activity, can influence glycation processes and potentially mitigate their role in AD progression. Diets high in refined sugars and processed foods are known to increase circulating AGEs, which contribute to oxidative stress, inflammation, and neuronal dysfunction in AD [24]. Conversely, dietary patterns rich in whole foods and antioxidants—such as the Mediterranean or DASH diets—have been shown to reduce systemic AGE levels and improve cognitive outcomes. While direct research linking aerobic exercise to reduced AGE accumulation in AD patients is limited, studies have shown that physical activity can mitigate factors contributing to AGE formation. For instance, aerobic exercise improves insulin sensitivity and glucose metabolism, which may, in turn, reduce AGE production. Additionally, exercise has been found to decrease systemic inflammation and oxidative stress, both of which are associated with AGE accumulation [25].

There is a significant relationship between serum and brain AGE levels, with the highest concentrations in participants suffering from T2D and AD [26]. This finding highlights the potential impact of AGEs on AD pathogenesis, particularly in the superior temporal gyrus, a region affected early in the disease. The findings suggest that adopting a low-AGE diet could reduce both circulating and brain AGEs, potentially lowering the risk of AD-related dementia in individuals with T2D and possibly the elderly population at large.

AGE accumulation is a shared pathological feature in both sporadic and familial AD, though it differs in timing and distribution. In sporadic AD, AGEs build up gradually with aging and metabolic stress, primarily in the cortex and hippocampus. In contrast, familial AD exhibits an earlier and more aggressive accumulation, including in less typical regions such as the striatum. These patterns underscore the role of systemic metabolic health in late-onset AD and support glycation as a potential therapeutic target. Moreover, the shared involvement of the AGE–RAGE axis across both forms of AD suggests a convergent pathological mechanism, offering opportunities for biomarker development and combined therapeutic strategies [27].

The role of AGEs in AD is further complicated by their ability to modify Aβ, facilitating its aggregation and promoting plaque formation. AGE modification of Aβ might be either a primary pathogenic event or a secondary consequence of Aβ deposition [28]. Although initial therapies targeting Aβ consistently failed to yield promising results, recent successful clinical trials have restored optimism in targeting Aβ as a viable strategy for AD treatment [29]. Immunopathological studies show that all plaque types in AD exhibit AGE immunoreactivity, suggesting that AGEs may also modify other amyloid-associated or unidentified proteins. This interaction exacerbates oxidative stress and inflammation, further contributing to neuronal damage in AD and other neurodegenerative diseases.

Recent studies have begun to elucidate the mechanical links between AGE-RAGE signaling and AD pathology. For example, a study utilized Sequential Window Acquisition of All Theoretical Fragment Ion Spectra (SWATH-MS) proteomics to show that AGE treatment in neuro2a cells upregulated lysosomal proteins implicated in AD, such as cathepsin B, asparagine endopeptidase (AEP), and acid ceramidase [30]. This upregulation was associated with increased production of Aβ1–42 and tau phosphorylation, which are key hallmarks of AD. The study suggests that inhibiting components of the AGE-RAGE axis could open new therapeutic possibilities for treating tau-related neurodegenerative disorders.

Furthermore, several biomarkers have emerged as promising tools for evaluating the effects of metabolic interventions on the progression of AD. Blood-based biomarkers such as phosphorylated tau (p-tau), Aβ, and neurofilament light chain (NfL) provide accessible indicators of core AD pathology and neuroaxonal damage, and their levels have been shown to respond to therapeutic modulation [28, 31]. Imaging modalities also offer valuable insights: fluorodeoxyglucose positron emission tomography (FDG-PET) can assess cerebral glucose metabolism, a parameter directly influenced by metabolic interventions, while structural magnetic resonance imaging (MRI) enables monitoring of brain atrophy and neurodegeneration over time [32]. Metabolic biomarkers such as the triglyceride-to-HDL cholesterol (TG/HDL-C) ratio and apolipoprotein A1 (ApoA1) levels have also been linked to cognitive decline and insulin resistance, making them useful indicators of systemic metabolic health in relation to AD [33]. Collectively, these biomarkers hold promise for tailoring and monitoring therapeutic strategies aimed at slowing neurodegenerative progression through metabolic pathways.

## Glycation in Alzheimer’s Disease

**Fig. 1:**
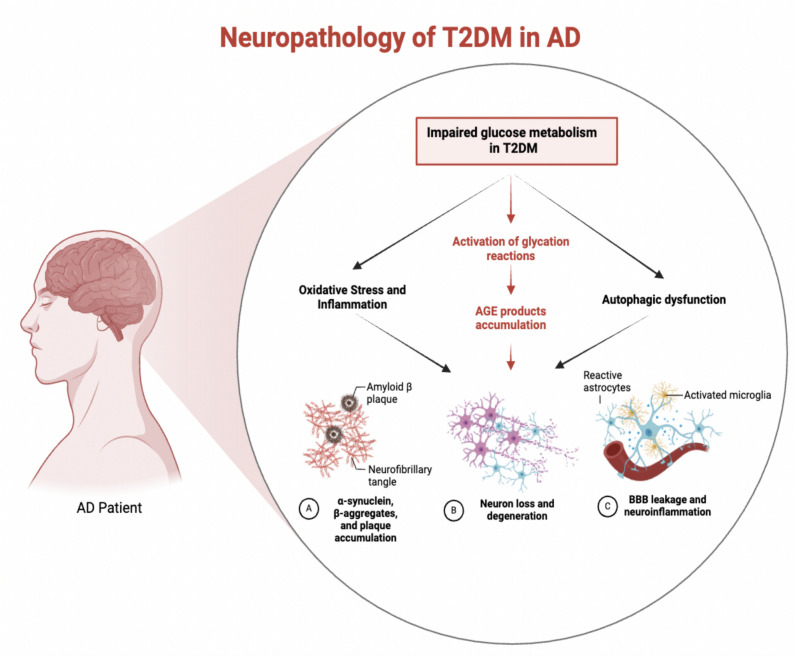
Diabetes increases glucose and reactive metabolites, accelerating AGE formation. The impaired metabolism also increases reactive oxygen species production, leading to oxidative stress and inflammation. It also disrupts cellular energy balance, leading to autophagy dysfunction. AGE product accumulation induces oxidative stress and inflammation, damaging neurons, and the blood–brain barrier (BBB), leading to leakage and further neuroinflammation

Glycation, driven by the Maillard reaction, is a non-enzymatic process that forms advanced AGEs by reacting sugars with proteins, lipids, or nucleic acids. AGEs have garnered significant attention for their role in aging and chronic diseases, particularly AD. The accumulation of AGEs has been implicated in AD pathology, especially in promoting Aβ aggregation, tau hyperphosphorylation, and neuroinflammation [34, 35]. Research suggests that AGEs contribute to the acceleration of Aβ aggregation in AD, amplifying neurodegeneration (Fig. 1) [36]. Glycation, a non-enzymatic modification of proteins, accelerates with elevated glucose and reactive carbonyl levels. In effect, glycation alters a protein’s charge and conformation, making it prone to misfolding and aggregation. For example, AGE adducts on lysine break salt-bridges and disrupt native structure [37]. Metabolically, conditions like hyperglycemia or oxidative stress elevate methylglyoxal levels [38] and age-related decline in glyoxalase detoxification further accelerates AGE accumulation [39]. In healthy aging, moderate glycation occurs due to intact detoxification systems, although glyoxalase I/II activity declines with age [39]. In AD, impaired brain glucose metabolism and insulin signaling, combined with inflammation, further inhibit detox pathways, leading to greater AGE accumulation, particularly around Aβ plaques and tau tangles. In diabetes mellitus, chronic hyperglycemia drives the highest glycation rates, markedly increasing HbA1c and systemic AGE burden. Serum pentosidine levels illustrate a gradient: healthy controls < AD < diabetes. Overall, glycation progresses slowest in aging, faster in AD, and fastest in diabetes, with RAGE amplifying its pathological effects across these states. Experimental models studying glycation in AD mainly focus on the accumulation of AGEs and their interaction with RAGE, both implicated in AD pathology. Transgenic models, such as 3xTg-AD mice, show increased RAGE expression in neurons and astrocytes, especially in aged individuals, mirroring elevated RAGE levels in human AD brains. This upregulation is linked to co-localization of RAGE with intracellular Aβ and tau, suggesting a role in promoting neurodegeneration. Dietary models using high-AGE diets have shown worsened memory deficits and increased hippocampal Aβ accumulation in AD-like mice, further supporting the link between AGEs and AD pathology. While these models replicate aspects of human AD, such as AGE accumulation, RAGE activation, and neuroinflammation, they do not fully capture the complexity of human disease due to species differences and the multifactorial nature of AD. Thus, while valuable for mechanistic studies, caution is needed when extrapolating findings to human disease [40].

### Glycation and Amyloid-Beta Aggregation

The interaction between glycation and Aβ is critical in understanding the progression of AD. Aβ oligomerization, a key AD feature, is accelerated by glycation, leading to increased amyloid plaque formation. High glucose concentrations have been shown to enhance the formation of AGEs, which in turn influence Aβ aggregation and stability. Glycated Aβ is more prone to forming toxic oligomers and amyloid fibrils, exacerbating neuronal toxicity [41]. These modified Aβ species resist proteasomal degradation, contributing to plaque accumulation [42].

Interestingly, glycation at specific lysine residues of Aβ destabilizes the fibrillar structure, promoting the formation of toxic soluble oligomers rather than stable fibrils. This shift is particularly concerning, as soluble oligomers are believed to be more neurotoxic than amyloid fibrils, contributing significantly to cognitive decline in AD [43].

### RAGE and the AGE-Amyloid Axis

RAGEs play a pivotal role in mediating the toxic effects of AGEs and glycated Aβ in AD. RAGE facilitates the transport of Aβ across the blood–brain barrier, promoting amyloid plaque formation while exacerbating neuroinflammation and oxidative stress [44, 45]. This interaction establishes a detrimental cycle of AGE accumulation, Aβ aggregation, and neuronal damage, with RAGE activation triggering further pathological processes such as tau hyperphosphorylation through the glycogen synthase kinase-3 (GSK-3) pathway, contributing to neurofibrillary tangle formation and AD progression [46, 47].

RAGE activation also induces oxidative stress and inflammation via the production of reactive oxygen species (ROS) and the activation of NF-κB, which releases inflammatory cytokines and cellular apoptosis, further compromising neuronal health [48]. Since RAGE expression is elevated in AD brains, therapeutic strategies targeting the AGE-RAGE axis are promising for mitigating disease progression. Soluble RAGE (sRAGE), which acts as a decoy receptor, competes with membrane-bound RAGE for binding to Aβ and AGEs, potentially reducing inflammation and neuronal damage [48].

### Oxidative Stress Role in Neurodegeneration

Oxidative stress plays a central role in the pathophysiology of T2DM and AD. In T2DM, hyperinsulinemia and impaired glucose metabolism increase NADH, FADH₂, and ATP levels, driving ROS production. Elevated ROS promotes non-enzymatic glycation and AGE formation, which activate RAGE—a pathway implicated in AD progression [49]. ROS also disrupts neuronal glucose metabolism by damaging key enzymes in glycolysis and the TCA cycle, reducing ATP synthesis [50]. In APP/PS1 models, metabolomics and redox proteomics have revealed widespread metabolic dysfunction, oxidative damage, and hyperglycemia, alongside oxidation of ATP synthase, aldolase, and α-enolase—proteins critical for energy homeostasis and synaptic function [51]. These impairments parallel AD features such as synaptic loss, Aβ deposition, and NFT accumulation. Additionally, T2DM exacerbates CNS insulin resistance and early Aβ accumulation, triggering a feedback loop of ROS, inflammation, and further IR [52]. This bidirectional relationship underscores oxidative stress as a shared driver of T2DM and AD, offering a potential therapeutic target.

### Glycation and Tau Hyperphosphorylation

Glycation amplifies neurodegeneration and cognitive decline in AD. Cross-linked AGE-modified Aβ and tau aggregates accumulate and resist clearance, disrupting proteostasis and accelerating synaptic loss [53]. Chronic AGE–RAGE signaling in neurons and glia sustains oxidative stress, inflammation, and tau hyperphosphorylation through NF-κB and kinases such as GSK-3β [36, 54]. Experimental models show that blocking AGE formation or RAGE activation with agents like aminoguanidine preserves synaptic proteins and rescues memory deficits [36, 55]. Clinically, conditions that elevate glycation, including aging and diabetes, roughly double the risk of developing AD, reinforcing the pathogenic role of AGEs in disease progression.

Glycation promotes tau hyperphosphorylation through RAGE-mediated activation of GSK-3, leading to NFTs [35]. NFTs, composed of hyperphosphorylated tau, disrupt neuronal microtubule dynamics and are closely linked to synaptic dysfunction and cognitive decline in AD [56].

AGEs colocalize with tau in AD brain tissues, amplifying oxidative stress and enhancing tau aggregation. This interaction further destabilizes neuronal function, promoting the progression of AD (Chaudhuri et al., 2018). The combined impact of AGE-induced Aβ aggregation and tau hyperphosphorylation underscores the importance of glycation in driving neurodegeneration. AGEs contribute to AD pathology by disrupting insulin signaling and promoting oxidative stress, both of which drive tau hyperphosphorylation and Aβ accumulation. In T2DM, AGEs impair insulin pathways and reduce PP2A activity [57], a key regulator that normally prevents tau hyperphosphorylation. This dysfunction—worsened by hypothermia commonly observed in T2DM—leads to tau misfolding and aggregation into NFTs [58]. These aggregates resist autophagic clearance, causing intracellular buildup, ROS generation, and mitochondrial dysfunction. The resulting oxidative stress triggers apoptotic pathways and synaptic degeneration, both critical in AD progression. Additionally, insulin resistance affects MAPK and AKT signaling [59] further destabilizing tau. Insulin oligomers accumulate in neurons with hyperphosphorylated tau, a common feature in AD and other tauopathies [60]. Diabetic AD models and CSF data reveal distinct tau phosphorylation patterns, especially in APOE ε4 carriers [61]. Elevated tau levels and reduced Aβ1–42 have also been observed in individuals with both T2DM and cognitive impairment, reinforcing the link between AGEs, tau, and Aβ pathologies [62].

### The Mechanistic Link Between AGEs, T2DM, and AD

The pathological link between T2DM, glycation, and AD involves impaired insulin signaling, hyperglycemia, and inflammation—all of which drive neurodegeneration. In T2DM, brain insulin resistance disrupts PI3K/AKT/GSK-3β signaling, leading to altered APP processing, increased Aβ accumulation, and tau hyperphosphorylation—core features of AD [63, 64]. Reduced insulin transport across the BBB, driven by systemic IR, aging, and inflammation, contributes to neuronal insulin deficiency and signaling failure. Hyperglycemia accelerates non-enzymatic glycation, resulting in AGE formation, which promotes protein misfolding and aggregation (Aβ, tau) and activates RAGE, triggering ROS and chronic neuroinflammation [65]. IR also impairs IDE activity, limiting Aβ clearance [66]. These changes, along with mitochondrial dysfunction, ceramide accumulation, and cytokine release, result in synaptic loss and cognitive decline. Together, these mechanisms underscore how T2DM and glycation contribute to AD progression and support targeting insulin-related and glycation pathways as potential therapeutic strategies.

### Biomarkers of Glycation in AD

Several glycation-related molecules have been proposed as potential biomarkers for AD. AGEs such as CML, CEL, and pentosidine accumulate in AD brains. Serum pentosidine levels are significantly elevated in AD compared to controls, and colocalize with Aβ plaques and tau tangles [67, 68]. CML is prominently detected in AD brain lesions and correlates with tau pathology. Circulating extracellular vesicle (EV) levels of carboxymethyllysine (CML) have been shown to distinguish early to moderate stages of Alzheimer’s disease. Within the brain, CML-modified neuronal proteins may spread intercellularly through EV-mediated transfer CML-modified proteins are more susceptible to oxidation and crosslinking, exacerbating neuronal dysfunction [69]. For instance, a small study reported detectable CSF pentosidine levels, which were decreased in AD compared to controls, suggesting altered AGE metabolism [70]. Notably, pentosidine levels distinguished AD patients from diabetic and age-matched healthy controls despite comparable renal function and were unrelated to conventional glycemic indicators such as HbA1c and fructosamine. This highlights pentosidine’s potential diagnostic value in Alzheimer’s disease beyond traditional markers of glycation [67]. In addition, elevated methylglyoxal promotes Alzheimer’s disease progression by enhancing AGE accumulation, oxidative stress, and tau hyperphosphorylation, ultimately driving neuronal dysfunction; thus, targeting MG detoxification and improving glucose metabolism may represent promising therapeutic strategies to reduce carbonyl stress and cognitive decline in AD [54]. Other circulating glycation markers, such as fructosamine, glycated albumin, and HbA1c, are also elevated in AD cohorts but lack specificity for distinguishing AD from DM. RAGE and its soluble form (sRAGE) have also been investigated as biomarkers. Plasma levels of RAGE ligands, including S100A12 (EN-RAGE) and HMGB1, correlate with dementia risk. A large cohort study (*n* ≈ 3890) found plasma RAGE ligands associated with prevalent dementia and skin AGE levels (measured by autofluorescence) correlated with cognitive impairment [71]. However, RAGE-based measures are not yet validated for diagnosis and are mainly explored as therapeutic targets. Experimental RAGE-PET imaging probes are under development for early detection [22].

Additional markers include TAGE and oxidative glycation products. Elevated MG-derived adducts, such as MG-H1, have been observed in AD, with age-related declines in Glo1/2 contributing to MG and AGE accumulation [39]. Although no glycation markers are part of routine AD diagnostics, they show promise for monitoring disease progression. Higher pentosidine levels have predicted faster cognitive decline [68]. However, no glycation biomarker is FDA-approved for AD, and they are currently under investigation as adjuncts to classical biomarkers (Aβ, tau) for early detection and tracking [67].

### Implications and Therapeutic Opportunities

The significance of glycation in AD highlights the critical role of metabolic health in neurodegeneration. Glycation is accelerated in conditions like T2DM, where chronic hyperglycemia increases AGE formation, leading to a heightened risk of AD. Chronic hyperglycemia increases the production of ROS, leading to oxidative damage in neurons. This stress environment contributes to both direct neuronal injury and activation of inflammatory pathways, setting the stage for neurodegeneration [72]. Elevated glucose levels promote the formation of AGEs, which trigger inflammation via the RAGE receptor. Human studies show that individuals with both T2DM and AD have the highest AGE levels, linking glycation stress to cognitive decline and brain inflammation [73]. T2DM-associated insulin resistance affects the brain’s ability to use glucose efficiently. This results in neuronal energy deprivation, impaired protective signaling, and increased vulnerability to AD pathology—supporting the idea of AD as a “type 3 diabetes” [74]. Together, oxidative stress, AGEs, and impaired metabolism drive chronic neuroinflammation. This leads to microglial activation, cytokine release, and ultimately neuronal and synaptic loss. Imaging studies confirm greater brain atrophy in individuals with T2DM, linking diabetes to accelerated neurodegeneration in AD [75].

Targeting the glycation process, particularly the AGE-RAGE axis, offers promising therapeutic potential for mitigating AD progression. Lifestyle interventions that reduce AGE formation, such as dietary modifications, improved glycemic control, and RAGE inhibitors, may provide viable strategies for slowing cognitive decline and synaptic dysfunction in AD [76, 77]. Further research into glycation’s role in AD, particularly its impact on Aβ and tau pathology, is crucial for developing targeted therapies to restore proteostasis, reduce oxidative stress, and prevent neuroinflammation.

#### Enzymatic Detoxification Pathways and Therapeutic Benefit

Cells deploy multiple enzymatic systems to detoxify glycating intermediates and reverse early glycation adducts. The glyoxalase pathway (glyoxalase I and II) is the primary defense against dicarbonyls, converting reactive species such as methylglyoxal (MG) into non-toxic D-lactate via glutathione-dependent catalysis [78]. Complementary enzymes also protect against glycation: aldo–keto reductases (e.g., aldose reductase) reduce MG and related α-oxoaldehydes to inert alcohols, and aldehyde dehydrogenases oxidize these aldehydes to acids like pyruvate [78]. Dedicated deglycation enzymes repair early glycation intermediates: fructosamine-3-kinase (FN3K) phosphorylates Amadori (fructosyl-lysine) residues, promoting spontaneous breakdown of the glycation adduct [79]. Similarly, the Parkinson’s protein DJ-1 (Park7) exhibits glyoxalase-like activity, consuming MG to form lactate [78]. In microbes, Amadoriases and related enzymes cleave early Schiff base/Amadori products; however, no mammalian amadoriase has yet been identified [79]. These enzyme systems collectively reduce the pool of reactive carbonyls and repair early glycation, thereby limiting downstream AGE accumulation.

#### Non-enzymatic Detoxification Strategies and Therapeutic Benefit

In addition to enzymatic defenses, non-enzymatic interventions can attenuate glycation. Small-molecule carbonyl scavengers like hydrazines or dipeptides directly trap reactive α-dicarbonyls before they form stable adducts, while general antioxidants (vitamins C/E, N‑acetylcysteine, etc.) inhibit free-radical–mediated glycoxidation steps. Metal-chelating compounds such as pyridoxamine sequester transition metals that catalyze sugar oxidation, further slowing AGE formation. Dietary polyphenols (flavonoids, catechins, stilbenes, etc.) combine several mechanisms: they directly bind methylglyoxal and other carbonyls, scavenge reactive oxygen species, and chelate metal ions, all of which impede AGE biosynthesis [80]. Lifestyle factors also play a role: for example, low-glycemic-index diets, calorie restriction and regular exercise reduce circulating glucose and oxidative stress, and minimizing intake of exogenous AGEs (through gentle cooking methods) has been associated with improved metabolic profile [81]. Together, these approaches lower systemic “carbonyl stress” and complement enzymatic defenses against AGE accumulation.

#### Experimental and Therapeutic Approaches and Therapeutic Benefit

Several pharmacological and experimental strategies have been developed to target glycation and AGE pathology. Aminoguanidine (a dicarbonyl-trapping agent) and benfotiamine (a lipid-soluble vitamin B₁ analog that activates transketolase) were early examples shown to inhibit AGE formation in diabetic models [78]. Compounds that cleave existing crosslinks have also been pursued: for instance, alagebrium (ALT-711) is a thiazolium derivative that breaks protein–AGE crosslinks, thereby restoring tissue elasticity and reversing glycation-induced arterial stiffnes. Blockade of AGE signaling is another strategy; small-molecule RAGE antagonists (e.g., FPS-ZM1, azeliragon) have been designed to prevent AGE–receptor interactions and blunt downstream inflammatory cascade [82]. More novel approaches include administering microbial AGE-degrading enzymes or probiotics that metabolize glycated compounds (Cohen, 2013) and gene therapies to enhance endogenous detoxification (e.g., overexpressing GLO1 in diabetic animals markedly lowered methylglyoxal and AGE levels [83]. Together, these experimental interventions—ranging from carbonyl traps and cofactor supplements to AGE breakers and biological therapies—aim to reduce AGE burden and mitigate glycation-related damage.

### Regional Vulnerability to Insulin Resistance and Metabolic Dysfunction in AD

Insulin-related cognitive decline often affects brain regions and functions similar to those impacted in AD, but it can emerge prior to the development of overt pathology. Memory deficits are particularly common, as the hippocampus and medial temporal lobe (MTL)—regions heavily reliant on insulin signaling for glucose uptake and synaptic plasticity—are among the first affected. Insulin resistance (IR) in late-middle age has been associated with reduced hippocampal volume and impaired episodic memory performance [84]. Even cognitively normal individuals with elevated IR demonstrate poorer verbal memory scores [85]. Executive functions and processing speed may also decline, with midlife hyperinsulinemia linked to accelerated deterioration in verbal fluency—a frontal-executive task—independent of amyloid burden [86]. Cross-sectional studies by Benedict et al. further showed that IR correlates with reduced grey matter (GM) volume in the temporal and frontal lobes and with lower verbal fluency in healthy older adults. These findings suggest that insulin dysregulation primarily affects memory, fluency, and attention, implicating the hippocampus and frontal cortex as key vulnerable regions. Importantly, such cognitive impairments can manifest in individuals without significant amyloid or tau pathology; for instance, patients with diabetes mellitus or prediabetes often experience mild cognitive deficits or “brain fog” driven by metabolic dysfunction rather than classical AD lesions. In individuals with established AD, comorbid IR appears to exacerbate global cognitive decline and brain atrophy beyond what would be expected based on amyloid or tau pathology alone [87]. Overall, insulin-related dysfunction seems to selectively impair memory circuits and associative cortical areas, paralleling the cognitive profile of AD but through distinct metabolic mechanisms.

## Deleterious Effects of AGEs

**Fig. 2:**
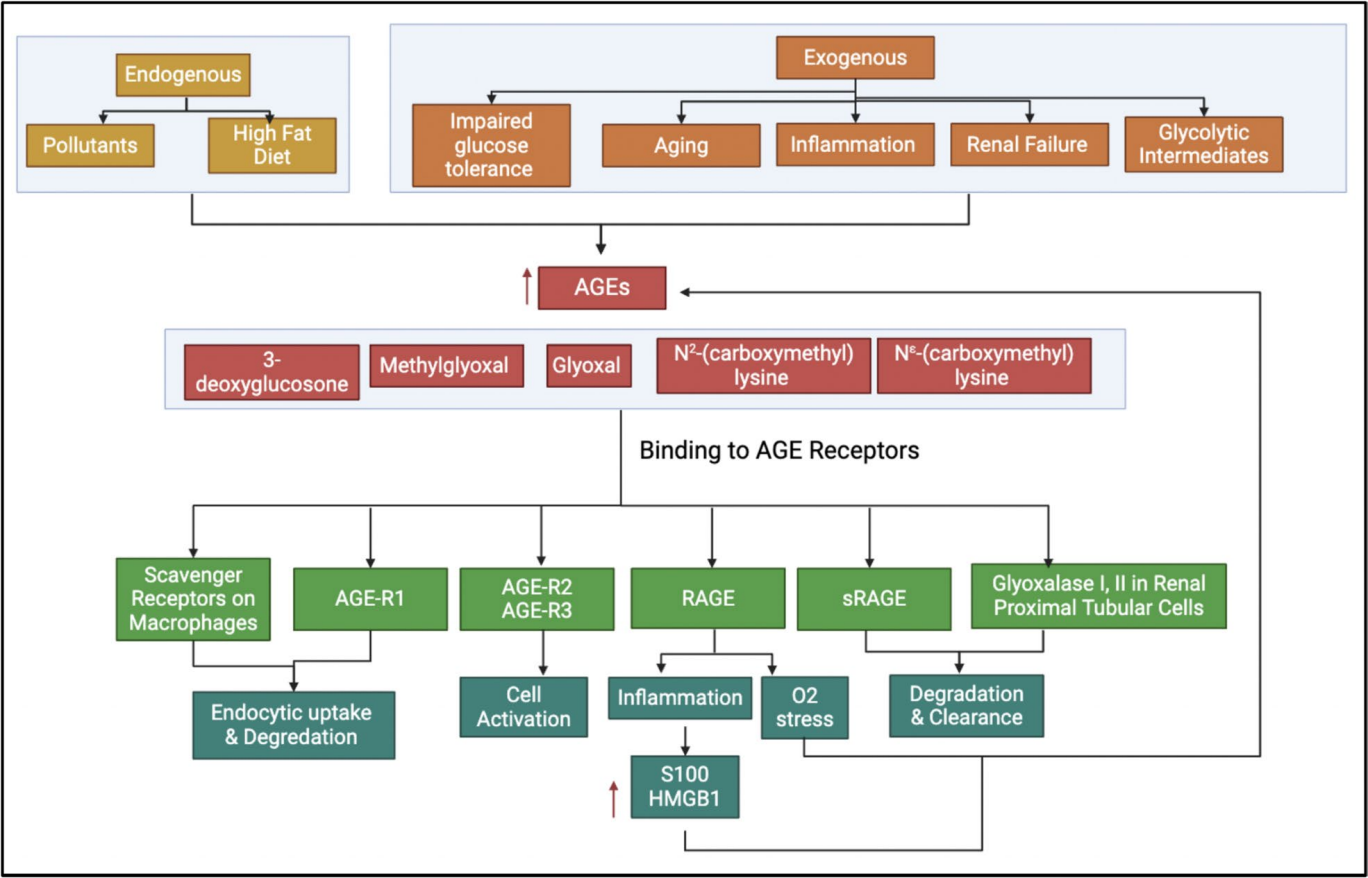
AGE formation and interactions with different RAGEs are due to the Maillard/glycation reaction. AGEs, which can be produced endogenously and exogenously via several methods, have different fates when binding to the AGE receptors. AGEs harm T2DM and AD by promoting inflammation, oxidative stress, and tissue damage. The accumulation of AGEs contributes to the pathogenesis of these conditions and represents a potential target for therapeutic intervention, highlighting the issue's complexity

Both intracellular and extracellular factors contribute to accumulating AGEs (Fig. 2). Notably, AGEs derived from dry-heat-cooked foods significantly elevate the risk of age-related diseases by RAGE, a key mediator in chronic inflammatory and degenerative conditions [88, 89]. Intracellularly, the breakdown of AGEs produces highly reactive intermediates known as mycotoxins, which can enter the bloodstream and damage large biomolecules, disrupting their natural integrity [10, 90, 91].

The adverse effects of AGEs are primarily chemical, pro-oxidant, and inflammatory, which can be categorized into two fundamental mechanisms. The first mechanism involves the direct impairment of protein function or extracellular matrix metabolism, independent of receptor binding [92, 93]. The second mechanism pertains to the interaction with RAGE, which activates signaling pathways such as MAPKs and PI3K, ultimately leading to the activation of NF-κB. Once activated, this transcription factor translocates to the nucleus and induces the expression of genes related to cytokines, growth factors, and adhesion molecules, including TNFα, IL-6, and VCAM-1. This cascade triggers chronic inflammation, vascular dysfunction, and an increased risk of atherosclerosis, thereby exacerbating neurodegenerative diseases such as AD [94–96].

Furthermore, NF-κB activation establishes a positive feedback loop that amplifies inflammatory responses, contributing to chronic conditions such as autoimmune diseases, cancer, and neurodegeneration. The interaction between AGEs and RAGE also stimulates NAD(P)H oxidase, leading to increased oxidative stress and further cellular damage. Understanding these processes is crucial, as they reveal the intricate connections between inflammation, oxidative stress, and disease progression, underscoring the urgency of advancing research in this field.

## The α-Dicarbonyl Detoxification and the AGE Degradation

Detoxifying α-dicarbonyls, such as methylglyoxal and glyoxal, presents a promising strategy for managing age-related diseases and reducing AGEs [46].The glyoxalase system, comprising enzymes like GLO1 and GLO3, plays a vital role in this detoxification process by significantly reducing AGE formation and thereby protecting against oxidative stress and inflammation [97, 98]. The effectiveness of this robust defense mechanism against AGE accumulation, especially in the context of age-related diseases characterized by low levels of GLO1 and glutathione, underscores the potential benefits of enhancing glyoxalase activity to lower AGE levels and mitigate AGE-related damage effectively.

Genetic studies have established links between polymorphisms in glyoxalase genes, particularly GLO1, and an increased risk of diabetic complications, such as nephropathy and retinopathy, in individuals with T2DM [99]. Specific single nucleotide polymorphisms (SNPs) in these genes correlate with reduced glyoxalase activity, highlighting the urgent need for effective detoxification strategies to mitigate these complications [100]. Furthermore, the DJ-1 gene, associated with PD, emphasizes the critical role of detoxification pathways in neurodegenerative conditions [101]. The involvement of DJ-1 in the oxidative stress response suggests that enhancing α-dicarbonyl detoxification could reduce cellular damage and lower the risk of PD and diabetes-related complications.

Moreover, enhancing α-dicarbonyl detoxification could improve the efficiency of the ubiquitin–proteasome system (UPS) and autophagy, both essential for degrading AGE adducts and maintaining cellular homeostasis. The UPS is a cellular pathway responsible for degrading and recycling unwanted proteins, while autophagy removes damaged or unnecessary cellular components. By boosting α-dicarbonyl detoxification, we can enhance the efficiency of these systems, thereby reducing AGE-related cellular damage and opening new avenues for therapeutic strategies targeting age-related diseases. This interconnectedness of detoxification processes highlights the importance of maintaining cellular health.

## Other Detoxifying Enzymes for α-Dicarbonyls

Before exploring the specific detoxifying enzymes for α-dicarbonyls, it is essential to highlight the protective role of NADPH-dependent aldo–keto reductases (AKRs). These evolutionarily conserved enzymes are critical for reducing harmful compounds [102]. Their absence has been linked to increased atherosclerotic lesions mediated by AGEs, underscoring their vital role in protecting against cardiovascular damage [103].

Another potential detoxification mechanism involves the sequestration of AGEs by lysozymes, which enhances renal excretion in mice and aids in the removal of AGEs [104]. The Nrf2 antioxidant pathway is also pivotal in regulating responses to oxidative stress and promoting the expression of detoxification enzymes that can potentially degrade AGEs. Upon exposure to oxidative stimuli, Nrf2 translocates into the nucleus and initiates the transcription of cytoprotective genes. These genes encode critical detoxification and antioxidant molecules, including enzymes involved in glutathione synthesis (γ-glutamyl cysteine ligase, GCLC, and GCLM), aldo–keto reductases (AKR1A1), and glyoxalase 1. This activation enhances cellular defenses against oxidative damage and improves the management of toxic metabolites like AGEs [105]. Fructosamine-3-kinase (FN3K) detoxifies fructosamines by phosphorylating low-molecular-mass and protein-bound fructose-lysines at the third carbon of their deoxyglucose moiety, resulting in the formation of fructosamine 3-phosphates. These phosphates decompose into inorganic phosphate and 3-deoxyglucosone, restoring the non-glycated amine. This process may help reduce the formation of AGEs, protecting tissues such as the kidneys, blood, and brain from glycation-related damage [91, 106]. Together, these detoxification mechanisms illustrate the body’s intricate and robust defenses against AGE accumulation, emphasizing their crucial importance in mitigating the harmful effects of AGEs on various organs and systems.

## Diabetes as a Risk Factor for AD

Research has established a significant link between T2DM and a 45–90% increased risk of dementia and AD, suggesting that AD may be a brain-specific form of diabetes, often referred to as Type 3 diabetes [107]. The development of both AD and T2DM is shaped by a dynamic interplay between genetic predispositions, environmental exposures, and metabolic dysfunction. Several shared genetic variants, including polymorphisms in CREBBP, MAPK, and the PI3K-AKT signaling pathways, have been implicated in the pathophysiology of both diseases, suggesting overlapping molecular vulnerabilities [108]. T2DM accelerates the formation of AGEs and enhances RAGE signaling, which modulates inflammation and reactive oxygen species generation. Studies in mouse models have demonstrated that diabetes can induce the formation of Aβ plaques and neurofibrillary tangles through tau hyperphosphorylation [109–111]. It is important to note that experimental models exploring the intersection of AD and T2DM primarily utilize approaches such as intracerebroventricular streptozotocin (STZ) injection, transgenic rodents, and high-fat diet-induced insulin resistance. The STZ model is particularly useful for examining cognitive deficits and insulin signaling impairments similar to those observed in sporadic AD, while transgenic models (e.g., APP/PS1, TgF344-AD) combined with metabolic interventions better represent the pathological synergy between familial AD features and metabolic disturbances. Despite their utility, these models have inherent translational limitations, including interspecies differences in metabolism and incomplete replication of human disease complexity, thereby moderating their predictive reliability for therapeutic efficacy [112].

### Production of AGEs in T2D

Recent research highlights the critical role of AGEs in inflammation and metabolic health. Elevated levels of AGEs are associated with increased inflammatory markers, independent of age, gender, body mass index (BMI), or smoking status [113]. This association extends to nondiabetic individuals after adjusting for BMI, waist circumference, and hemoglobin A1C, suggesting that AGEs may influence insulin resistance and overall metabolic health even in those without diabetes [114].

**Fig. 3:**
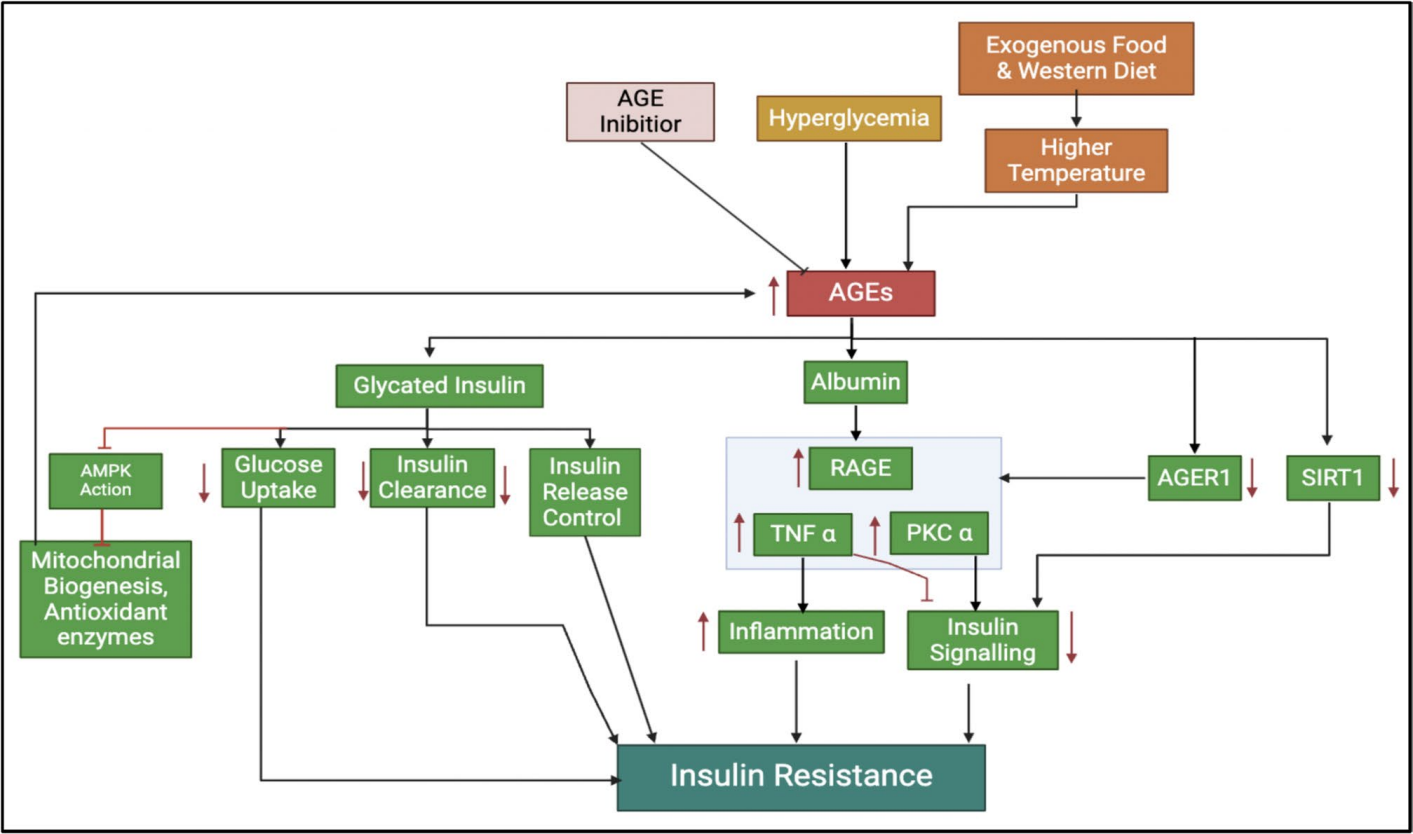
The relationship between AGE accumulation and insulin resistance. Our research has significant implications, as it reveals that AGEs can form glycated insulin and albumin through glycation processes involving glucose or reactive dicarbonyls, which modify these proteins and impair their function. This modification decreases SIRT1 and AGER1 levels by inducing oxidative stress and inflammation, suppressing their expression and activity. Consequently, glycated insulin leads to increased insulin resistance by impairing insulin’s ability to bind to its receptor and activate signaling pathways, disrupting glucose uptake and metabolism. These implications underscore the importance of further research in this area

Glycation affects insulin sensitivity through several mechanisms (Fig. 3). It modifies insulin at the phenylalanine residue on the beta chain, reducing its effectiveness by approximately 20% and necessitating higher doses for effective glucose management [115, 116]. Furthermore, glycation of proteins such as albumin elevates tumor necrosis factor-alpha (TNF-α) levels, which induces inflammation and suppresses insulin signaling [117, 118]. Additionally, methylglyoxal (MG), an intermediate in AGE formation, disrupts AMP-activated protein kinase (AMPK) function, impairing hepatic insulin metabolism and increasing lipogenesis and gluconeogenesis, thereby exacerbating insulin resistance [91]. These findings underscore glycation’s complex and significant role in insulin resistance and metabolic dysfunction.

### Insulin Signaling Impairment in AD Pathogenesis

Research reveals a strong link between changes in energy metabolism and AD development.

Brain insulin resistance and impaired insulin/IGF‑1 signaling have emerged as independent drivers of cognitive decline in AD, even aside from amyloid-β and tau pathology. In AD brains insulin/IGF signaling deficits mirror diabetes‐like dysfunctions across neurons, glia, and vasculature, leading to multifactorial damage [119]. For example, reduced insulin-mediated PI3K/Akt activity and unchecked GSK‑3β in insulin‑resistant neurons impair synaptic plasticity and neuronal survival [120]. These signaling disturbances also provoke chronic neuroinflammation, oxidative stress, and energy failure—manifesting as decreased glucose uptake, lower ATP production, synaptic weakening, and myelin loss [121]. Notably, brain regions rich in insulin receptors (such as the hippocampus and medial temporal lobe) show disrupted insulin signaling in AD, and the degree of hippocampal insulin resistance predicts worse cognitive performance [122]. Thus, a “type‑3 diabetes”–like state of brain insulin resistance can directly compromise neuronal metabolism, synaptic function, and plasticity, driving memory loss and dementia independently of Aβ/tau aggregates [120].

Furthemore, Aβ oligomers inhibit insulin signaling by blocking the auto-phosphorylation of insulin receptors on hippocampal neurons, contributing to impairments in learning, memory, and overall neurological function [123]. These oligomers also disrupt the tumor necrosis factor-alpha (TNF-α)/c-Jun N-terminal kinase (JNK) pathway, leading to neuroinflammation and further AD progression. By inhibiting insulin receptor substrate-1 (IRS-1), Aβ oligomers induce insulin resistance, which is linked to metabolic dysfunction and accelerates AD progression [124]. Insulin resistance exacerbates β-amyloid accumulation and plaque formation, creating a vicious cycle that promotes AD. Studies indicate that high-fat diets increase the levels of Aβ oligomers and result in memory impairment. Still, insulin injections can counteract these effects, underscoring the cyclical relationship between insulin resistance and Aβ oligomers [106]. Additionally, mice lacking insulin-degrading enzymes exhibit hyperinsulinemia, glucose intolerance, and elevated cerebral β-amyloid levels, further connecting insulin resistance to AD [125].

Peripheral and brain insulin resistance, while mechanistically related, represent distinct pathophysiological entities. Peripheral insulin resistance in T2DM affects classical insulin-responsive tissues such as muscle, liver, and adipose tissue, leading to systemic hyperglycemia and compensatory hyperinsulinemia. In contrast, brain insulin resistance refers to impaired insulin signaling within the CNS, which can occur independently of peripheral metabolic dysfunction. This central impairment disrupts neuronal glucose uptake, synaptic maintenance, and neuroplasticity, contributing to cognitive decline in AD [126, 127]. These distinctions complicate the therapeutic repurposing of antidiabetic agents for AD. One of the primary challenges is BBB permeability, as many systemic antidiabetic drugs—including insulin, GLP-1 analogs, and thiazolidinediones—have limited ability to cross into the CNS [128]. Intranasal insulin delivery has shown promise in overcoming this barrier while avoiding peripheral hypoglycemia. Moreover, achieving effective central drug concentrations may necessitate altered dosing regimens, which increases the risk of systemic adverse effects, particularly in older patients. The pharmacodynamics of these agents may also differ in the brain, potentially producing cognitive effects that vary with individual factors such as APOE genotype. For example, PPARγ agonists have shown cognitive benefit only in certain subgroups of AD patients [129]. These considerations highlight the need for precise delivery systems, dose optimization, and patient stratification when targeting insulin signaling pathways in AD. This interplay highlights potential therapeutic targets for addressing both metabolic dysfunction and neurodegeneration.

### Insulin Signaling and Tau Hyperphosphorylation

The tau protein, which is crucial for stabilizing microtubules in axons, becomes hyperphosphorylated due to impaired insulin signaling, leading to the formation of NFTs and subsequent neuronal degeneration in AD [130]. Increased activity of glycogen synthase kinase three beta (GSK-3β), resulting from reduced Akt signaling, promotes tau hyperphosphorylation and NFT formation [131]. Research suggests that inhibiting GSK-3β and enhancing insulin or insulin-like growth factor 1 (IGF-1) signaling can help regulate tau phosphorylation and prevent neurodegeneration [132]. The link between AD and T2DM is particularly notable due to impaired insulin signaling. Elevated GSK-3β activity in AD increases Aβ production and tau phosphorylation, exacerbating neuronal damage and synaptic impairments [20, 133]. Targeting insulin signaling and GSK-3β activity presents a promising strategy for treating neurodegeneration in both AD and related metabolic disorders.

### Glycation and Gut Microbes in AD and T2DM

**Fig. 4:**
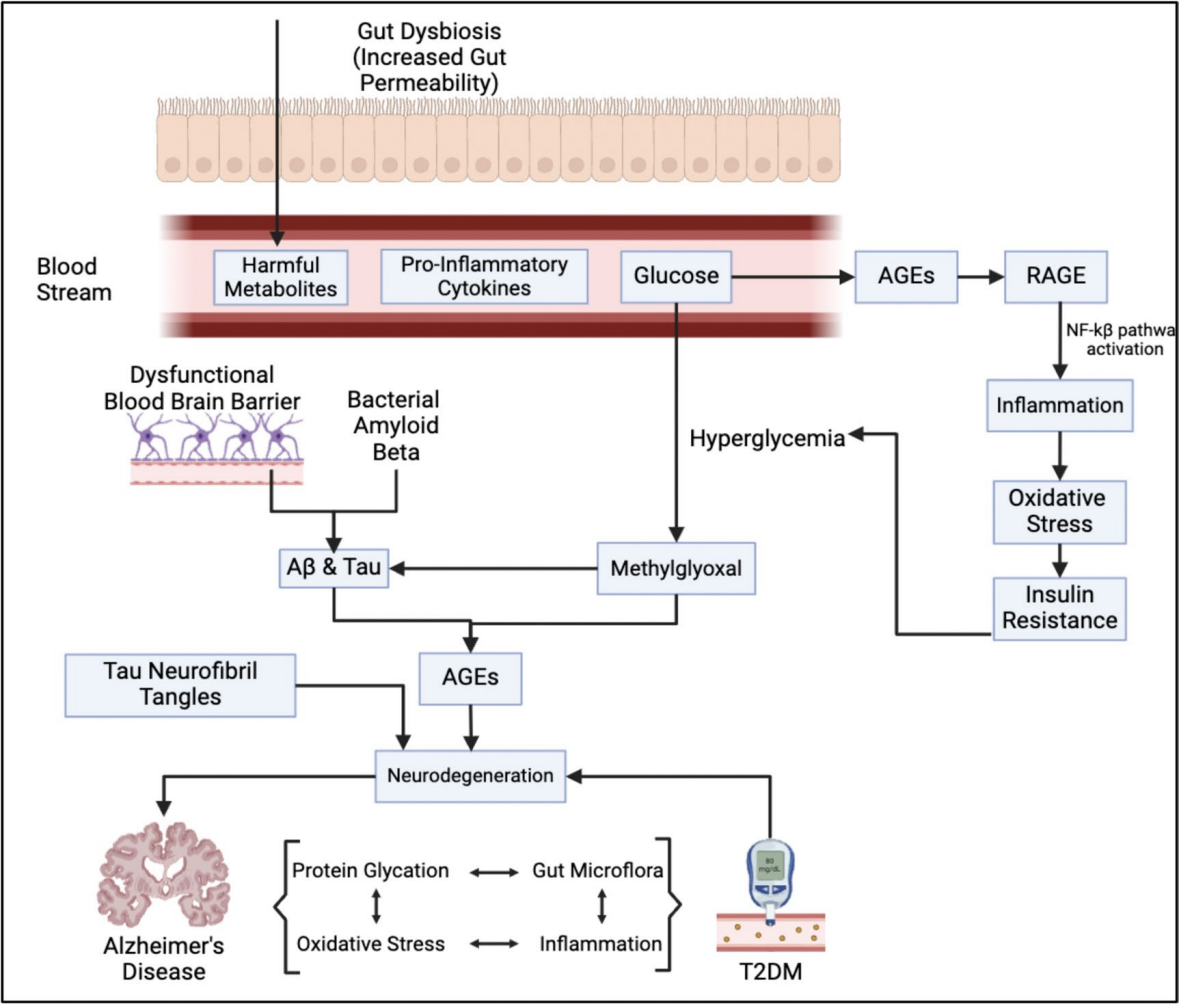
Glycation of gut proteins can disrupt the balance of gut microbiota, leading to increased gut permeability. This allows the entry of harmful byproducts that cause inflammation and the activation of various harmful pathways, including insulin resistance and Aβ and Tau buildup. These processes collectively contribute to the development and progression of T2DM and AD

Imbalances in the gut microbiome can significantly influence levels of AGEs and the progression of AD (Fig. 4). Certain gut bacteria convert dietary components into AGE precursors, and their dysbiosis can lead to inflammation and neurodegeneration. Maintaining gut health is crucial for managing AD, particularly by focusing on short-chain fatty acids (SCFAs), the impact LPS, and the effects of aging on microbial populations.

SCFAs, produced from dietary fibers, play a vital role in gut-brain communication, reducing neuroinflammation and supporting the integrity of the gut barrier. Other microbial metabolites, such as dopamine and serotonin, also contribute to brain health, underscoring the necessity of a balanced gut microbiota and sufficient fiber intake.

Microbial imbalances can increase gut permeability, allowing bacterial products like LPS to enter systemic circulation, leading to inflammation and enhanced AGE production and accumulation. Elevated levels of LPS and other microbial components have been observed in AD patients, linking gut health to cognitive impairment [134–137].

Shifts in gut bacteria associated with AGEs can exacerbate inflammation and damage the intestinal barrier, potentially accelerating the progression of AD [138]. Aging alters gut microbiota composition, increasing oxidative stress and mitochondrial dysfunction [139], while germ-free mice exhibit reduced oxidative stress [140]. Persistent gut inflammation resulting from aging can lead to neuroinflammation and compromise the blood–brain barrier [141, 142].

Microbiota transfer from aged to young rats impairs cognitive function while modifying gut microbiota in AD models can reduce disease markers. For instance, antibiotic treatment in 5xFAD mice decreased Lactobacillaceae, reduced plaque formation, and lowered RAGE expression [143]. These findings highlight the significant impact of gut microbiota changes on AGE accumulation and neurodegeneration, suggesting that dietary and probiotic interventions could improve mental health and mitigate age-related diseases.

Therapeutic modulation of the gut microbiota using probiotics (Lactobacillus, Bifidobacterium), prebiotics (e.g., inulin), and dietary fiber has shown promise in restoring microbial balance and mitigating inflammatory processes. Additionally, fermented foods such as kefir and pharmacologic agents like metformin have been linked to improved cognitive outcomes and favorable microbiome alteration. Twelve weeks of probiotic supplementation have been shown to improve oxidative stress markers, reduce inflammation, and enhance both quality of life and physical activity in individuals with mild to moderate Alzheimer’s disease [144] Furthermore, probiotic strains like *Lactobacillus acidophilus* have demonstrated antidiabetic effects and support intestinal health [145]. These findings support the potential of gut microbiota-targeted interventions as adjunctive strategies in managing metabolic and neurodegenerative disorders.

### Lifestyle as a Link Between AD and T2DM

AD and T2DM share several pathophysiological features, as T2DM—often associated with obesity and sedentary lifestyles—leads to chronic hyperglycemia that can damage the brain. Notably, both conditions exhibit insulin resistance, impaired glucose metabolism, and amyloid-beta aggregation, which has prompted some to label AD as “type 3 diabetes” or the “diabetes of the brain” [146–148]. Data from large-scale studies, including UK Biobank neuroimaging and cognitive assessments, reveal that T2DM is linked to deficits in executive function and processing speed, along with gray matter atrophy in regions such as the ventral striatum, cerebellum, and putamen—changes that mimic, yet appear earlier than, normal aging, with greater neurodegeneration associated with longer disease duration. Importantly, metformin treatment did not yield cognitive improvements, suggesting that mere glycemic control may be inadequate for preserving brain health [149]. Additionally, research involving 5653 participants demonstrated that diabetic individuals experienced a 45% faster decline in cognitive domains like memory and reasoning compared to a 29% decline in normoglycemic individuals, highlighting the significant role of both disease duration and glycemic control in accelerating neurocognitive decline [150].

### Dietary Intake Implications in AD and T2DM

Diet plays a crucial role in regulating glucose levels and supporting cognitive health in individuals with T2DM and AD. Both excessive caloric intake and overly restrictive diets can disrupt metabolic balance, but consistent dietary monitoring can improve glycemic control and may reduce neurodegeneration by limiting metabolic stress and AGE formation [2]. For elderly patients with both T2DM and AD, a less aggressive approach to glucose control—allowing slightly higher blood sugar targets—has shown better outcomes, possibly by minimizing oxidative stress from strict glycemic management [151].

In terms of diet, a balanced intake of macronutrients and low-glycemic index foods can help regulate glucose spikes and reduce AGE accumulation, which is crucial in managing both conditions [152]. The Mediterranean diet has demonstrated particular benefits, improving insulin sensitivity, supporting cardiovascular and cognitive health, and reducing AGE-related damage by emphasizing fruits, vegetables, legumes, whole grains, olive oil, and moderate fish and dairy intake, while limiting red meat [153]. The DASH and MIND diets have also shown promise in reducing AD risk, although the evidence is less extensive [154].

The ketogenic diet, by restricting carbohydrate intake and increasing fat consumption, may decrease endogenous AGE formation and improve mitochondrial function, reducing neuroinflammation and oxidative stress. These effects, along with improvements in weight, lipid profiles, and HbA1c, are beneficial for T2DM patients [155].

### Genetics

Recent genomic studies have begun to illuminate shared molecular underpinnings between T2DM and AD, offering new perspectives on risk stratification and targeted therapies. One study utilized non-negative matrix factorization (NMF) to analyze gene expression profiles and identified 241 differentially expressed genes (DEGs), including ACTB, SERINC3, and ZMIZ1, which are involved in immune-related pathways such as T cell activation and chemokine signaling—both of which are increasingly recognized in the pathology of T2DM and AD [156]. Similarly, another investigation applied NMF to cortical neuron datasets and, through protein–protein interaction analysis, highlighted overlapping gene networks involving CDKN1A, COL22A1, EIF4A, GFAP, SLC1A1, and VIM, further supporting the hypothesis of genetic convergence in these two diseases [157].

In parallel, integrative genome-wide association study (GWAS) analyses have provided complementary evidence. Using a conditional false discovery rate framework, Wang et al. [158] identified 78 AD-associated single-nucleotide polymorphisms (SNPs), many of which are also implicated in T2DM. Among these, genes such as TP53INP1, TOMM40, and NDUFAF6 emerged as key players in mitochondrial regulation and oxidative stress—pathways known to bridge metabolic dysfunction and neurodegeneration. These findings reinforce the notion of a shared genetic architecture and highlight novel targets for future interventions that address the metabolic-inflammatory axis linking T2DM and AD.

## A Dual Effect of Drugs in AD and T2D

**Fig. 5:**
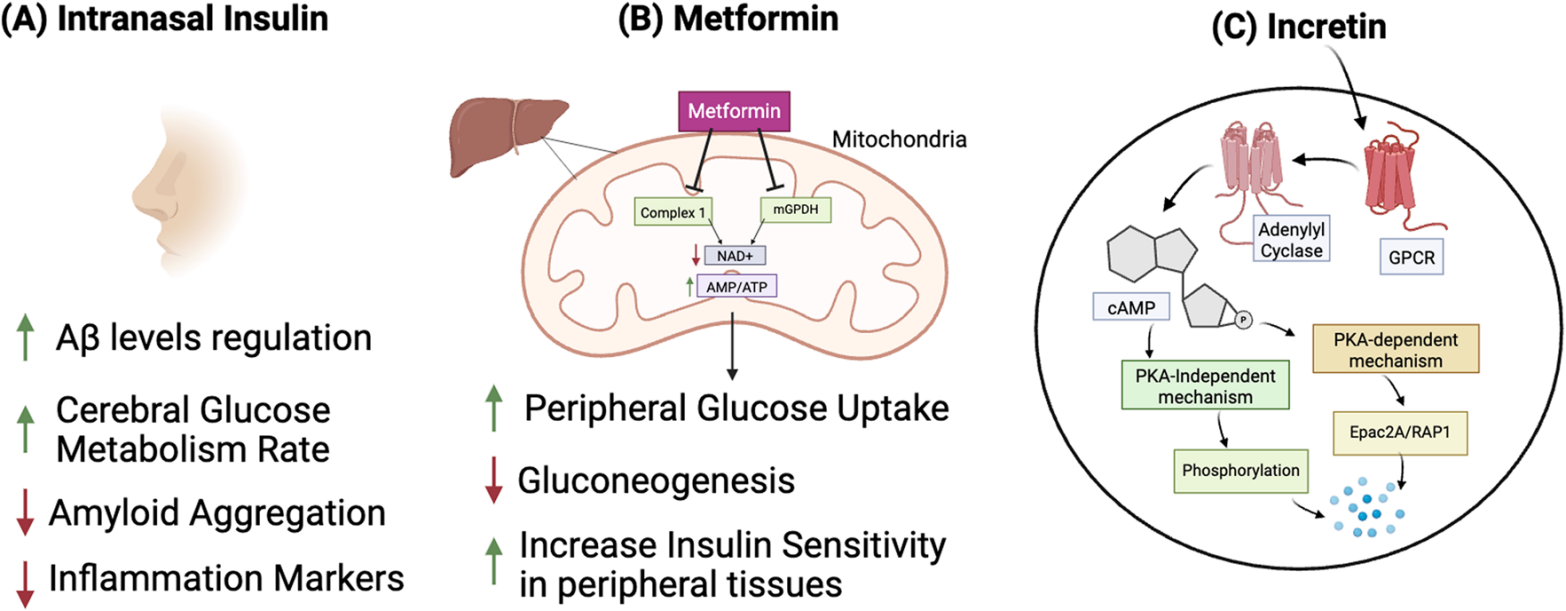
Antidiabetic drugs influence on AD. **A** Intranasal insulin bypasses the blood–brain barrier, regulating cerebral glucose metabolism and inhibiting AGE aggregation. **B** Metformin reduces glucose synthesis in the liver, enhances muscle insulin sensitivity, delays glucose absorption, and affects glucose metabolism by inhibiting mitochondrial complexes. It improves insulin sensitivity, reduces inflammation, enhances mitochondrial function, and promotes autophagy, thus inhibiting amyloid plaque formation. **C** Incretins activate the cAMP signaling pathway in pancreatic β-cells, affecting insulin exocytosis and granule preparation. They inhibit amyloid aggregation by enhancing insulin signaling, providing neuroprotection, reducing inflammation, modulating amyloid-processing enzymes, and improving mitochondrial function and autophagy

Recent studies have explored the potential therapeutic crossover between T2D and AD, particularly given their overlapping pathophysiological characteristics [126]. An intriguing hypothesis posits that antidiabetic medications, typically used to manage T2DM, may also benefit treating AD [159]. Originally designed to manage blood glucose levels, antidiabetic medications have also shown promise in protecting neural health by modulating inflammation, reducing oxidative stress, supporting mitochondrial activity, and influencing overall energy metabolism in the brain [160]. Clinical evidence suggests improvements in cognitive function among AD patients treated with antidiabetic medications such as intranasal insulin, metformin, thiazolidinediones, and incretins [161]. These drugs, which affect metabolic pathways, are being investigated for their potential dual benefits in addressing cognitive decline and insulin resistance (Fig. 5).

### Modulation of the Glycation Pathway

Most anti-diabetic drugs show mechanistic promise for reducing glycation stress. Preclinical studies consistently show metformin, GLP-1 agonists, DPP-4i, SGLT2i, and TZDs suppress AGE/RAGE signaling, oxidative stress, and neuroinflammation [162]. Observational cohorts suggest that GLP-1RAs, SGLT2i, and DPP-4i are associated with lower dementia incidence in T2D patients [163]. Small trials report cognitive benefits with liraglutide or intranasal insulin [39]. However, large RCTs are sparse or negative: exenatide and TZD trials showed no benefit [164], and intranasal insulin trials have mixed results [165]. Thus, despite strong mechanistic rationale, clinical evidence for AD prevention/treatment with these drugs remains preliminary. Anti-diabetic drugs may have a “dual” effect on diabetes and AD by mitigating glycation-related toxicity. Mechanistic studies indicate that many classes (metformin, incretin therapies, SGLT2 inhibitors, TZDs) attenuate AGE formation or RAGE activation, thereby reducing downstream inflammation and neurodegenerative processes [166]. Some human data support cognitive benefits (especially for GLP-1 agonists and perhaps SGLT2i [167]. Nevertheless, definitive proof in AD populations is lacking. Future work should fill gaps with well-designed trials of these agents in AD and clarify how much of their neuroprotective effect comes from direct anti-glycation actions versus general metabolic control.

### Intranasal Insulin and Its Benefits for AD Cognitive Improvement

Insulin, a hormone produced by the pancreas, regulates blood glucose levels by facilitating glucose uptake into cells for energy production or storage. In the context of AD, insulin treatment has been shown to mitigate the harmful effects of proteins such as Aβ and tau, which are critical contributors to the formation of amyloid plaques and neurofibrillary tangles—hallmarks of AD pathology. By modulating these proteins, insulin may reduce their aggregation and toxicity, potentially alleviating some neurodegenerative processes associated with the disease [168].

Research indicates that increased insulin levels can enhance memory in AD patients, even without high blood sugar, highlighting insulin’s role in cognitive function [169, 170]. Intranasal insulin delivery, which bypasses the risk of hypoglycemia, has been shown to rapidly reach key brain areas, such as the cortex and hippocampus, within 15–30 min [171]. A pilot study involving 24 patients with mild cognitive impairment (MCI) or early AD demonstrated that a 3-week intranasal insulin intervention improved working memory and cognitive skills [172]. Furthermore, a more extensive study with 104 patients experiencing MCI or mild to moderate AD reported cognitive and functional improvements after 4 months of chronic intranasal insulin administration [128]. These improvements were associated with changes in β-amyloid levels and the cerebrospinal fluid (CSF) β-amyloid/tau protein ratio, suggesting that intranasal insulin may be a viable therapeutic approach for AD without significant side effects.

A recent study investigating the effects of intranasal insulin detemir on patients with MCI and AD revealed enhancements in cognitive function, including verbal and audiovisual memory [169]. However, the study noted that insulin’s effects on cognitive function may vary depending on the ApoE genotype. Specifically, insulin appeared to benefit AD patients lacking the ApoE4 allele, suggesting a potential therapeutic advantage for this subgroup. Conversely, insulin administration could be less effective or even exacerbate symptoms in patients with the ApoE4 allele. This indicates the need for genotype-specific approaches when considering insulin treatment for cognitive decline in AD [173].

In summary, insulin therapy, mainly through intranasal delivery, holds promise for enhancing cognitive function and modulating harmful protein activities in AD. However, its efficacy may be influenced by genetic factors, such as the presence of the ApoE4 allele, emphasizing the importance of personalized treatment strategies.

### Metformin in AD

Metformin is commonly prescribed for T2DM due to its effectiveness in managing hemoglobin A1c levels, promoting weight management, and reducing cardiovascular risk, making it a preferred initial treatment choice [174]. Metformin reduces hepatic glucose production, improves insulin sensitivity, delays glucose absorption, and inhibits mitochondrial functions, lowering NAD + and ATP levels. Long-term use of metformin has yielded mixed results: Infante linked it to cognitive impairment, potentially due to vitamin B12 deficiency [175]. Ng found that it reduced the risk of cognitive decline after four years [43]. Notably, metformin use in T2DM patients is associated with a 24% reduction in the risk of dementia compared to those not on antidiabetic medications [176]. It has shown benefits for executive function, memory, and attention, although it does not appear to affect cerebrospinal fluid AD biomarkers [177]. Some studies suggest a slight increase in AD risk with long-term metformin use, possibly related to vitamin B12 deficiency [177]. Overall, while metformin is effective for managing T2DM, its effects on cognitive function and dementia risk remain complex and warrant further investigation.

### GLP-1 Agonists (Incretin) in AD

Glucagon-like peptide-1 (GLP-1) and glucose-dependent insulinotropic polypeptide (GIP) are incretins that stimulate insulin secretion, enhance beta-cell proliferation, and regulate glucose levels [178]. GLP-1, in particular, may reduce RAGE expression, thereby preventing inflammation and AGE accumulation. GLP-1 binds to its receptor (GLP-1R) in various tissues, activating the cAMP pathway to increase insulin release, inhibit glucagon secretion, and slow gastric emptying [179]. This mechanism also modulates inflammatory responses and reduces oxidative stress.

By decreasing RAGE expression, incretins can prevent chronic inflammation and tissue damage caused by AGEs, highlighting GLP-1’s potential in managing T2DM complications. Despite promising results in animal studies, current research has not demonstrated a reversal of AD pathology in humans. Further studies are needed to clarify the effects of incretins on AD across different stages in human subjects.

### Neuroprotective Mechanisms of Antidiabetic Agents Beyond Glycemic Control

Neuroinflammatory processes are increasingly recognized as central drivers of neurodegeneration. In Alzheimer’s and Parkinson’s diseases, prolonged activation of central immune responses, particularly involving microglia and pro-inflammatory cytokines, contributes to progressive neuronal loss. Some antidiabetic drugs have been shown to counteract these effects by dampening key inflammatory signals [180]. Thiazolidinediones and similar agents are capable of downregulating major inflammatory mediators, including TNF-α and IL-6—cytokines strongly implicated in neurodegenerative progression. This cytokine suppression may help interrupt chronic inflammatory cycles in the brain and protect vulnerable neurons from immune-mediated injury [181].

The ability of antidiabetic drugs to influence microglial polarization has also emerged as a relevant mechanism. By encouraging a shift from pro-inflammatory (M1) to anti-inflammatory (M2-like) microglial states, these agents may reshape the neuroimmune landscape toward one more conducive to repair and regeneration [182]. Together, these anti-inflammatory actions suggest that certain antidiabetic medications could serve dual roles: metabolic regulators and modulators of harmful neuroinflammation.

Neurons rely heavily on mitochondrial function for energy production, redox balance, and calcium regulation. Impairments in these systems are common in neurodegenerative disorders. Antidiabetic drugs have been reported to support mitochondrial health through multiple mechanisms [183]. Certain agents, particularly those that activate the PGC-1α signaling axis, have been shown to promote the synthesis of new mitochondria. This biogenic effect enhances both the quantity and efficiency of mitochondria within neurons, supporting their metabolic demands [184]. By improving mitochondrial respiration and ATP synthesis, antidiabetic medications contribute to a more stable and resilient neuronal energy supply. These improvements help neurons maintain function in the face of metabolic stress and may limit progression of energy-deficiency-driven neurodegeneration [185]. In essence, the ability of these agents to stabilize mitochondrial performance offers an additional layer of protection against the cellular dysfunctions underlying neurodegenerative diseases.

### Implications of Repurposing T2DM Treatment for AD

Despite growing interest in repurposing antidiabetic medications for the treatment of AD, several important limitations and risks must be considered. Agents such as GLP-1 receptor agonists and SGLT2 inhibitors have shown neuroprotective effects in T2DM populations; however, their efficacy in non-diabetic AD cohorts remains inconclusive, as most clinical data are drawn from diabetic patients [186]. Furthermore, the heterogeneity of AD pathology presents a challenge—these drugs primarily address insulin resistance and metabolic dysfunction, but may not directly influence Aβ or tau-related pathways. Trials investigating agents like metformin or intranasal insulin have yielded mixed results, with some cognitive benefits observed, while others show limited impact [186]. Safety concerns also exist, particularly in older adults with cognitive impairment. TZDs have been associated with fluid retention and weight gain, while sulfonylureas and insulin increase the risk of hypoglycemia, which can be detrimental in cognitively vulnerable individuals [161]. Additionally, polypharmacy in AD populations raises the risk of drug–drug interactions.

Similarly, targeting glycation pathways—another shared feature of AD and T2DM—must be approached with caution. While AGE inhibition has therapeutic potential, some agents such as aminoguanidine have shown hepatotoxicity and gastrointestinal side effects in trials [187]. Excessive suppression of AGEs may interfere with normal physiological roles in immune regulation and tissue repair [188], and chelating anti-glycation compounds may impair micronutrient absorption. Furthermore, modulating glycation could alter drug metabolism or interact with concurrent medications, complicating polypharmacy regimens. Natural compounds like polyphenols offer a safer alternative but often suffer from low bioavailability and uncertain long-term effects [80]. Together, these observations highlight the importance of designing targeted, selective interventions with rigorous clinical validation to ensure safety and therapeutic value in AD populations.

### Clinical Trials and Anti-glycation Therapies

Targeting glycation in neurodegeneration presents significant challenges. AGE formation is non-enzymatic, largely irreversible, and chemically heterogeneous, making it difficult for inhibitors to comprehensively block their formation [22]. Anti-AGE drugs, such as aminoguanidine, have shown safety concerns, including liver dysfunction and vasculitis at higher doses [189]. Effective CNS delivery is another obstacle, as many AGE inhibitors and detoxifying agents have poor brain penetration, and RAGE blockade may interfere with its normal functions. Clinical failures, such as the Phase III trial of azeliragon, further highlight these barriers. Moreover, glycation damage accumulates slowly over time, meaning significant injury may already be established by the time AD is diagnosed. Thus, despite its promise, anti-glycation therapy faces substantial biological and translational hurdles (Pasten, 2021).

The oral small-molecule inhibitor azeliragon (TTP488, PF-04494700) was the lead RAGE inhibitor anti-glycation drug tested. A Phase 2 safety trial (10 weeks) found azeliragon was well-tolerated but produced no change in amyloid or cognition (Sabbagh, 2011). A larger multicenter Phase 2 trial (*n*≈399, 18 months) evaluated low vs. high doses; the high dose was dropped early due to safety issues and no cognitive benefit, and the low dose showed futility (Galasko, 2014). Ultimately, the 18-month Phase 3 “STEADFAST” trial (880 mild AD patients, 5 mg/day vs. placebo) was terminated early in 2018 after failing both primary cognitive endpoints. A follow-up Phase 2/3 trial in AD patients with type-2 diabetes (targeting those with high HbA1c) was also halted in 2020 for lack of efficacy. In sum, RAGE blockade with azeliragon did not slow AD progression; high-dose azeliragon even seemed to accelerate decline. These negative results led Pfizer/vTv to stop development of RAGE inhibitors for AD.

Thiamine-derived anti-glycation (Benfotiamine): Benfotiamine is a high-bioavailability vitamin B1 analog that shunts glycolysis intermediates away from AGE formation. In a 12-month Phase IIa trial (71 amyloid-positive subjects with MCI/mild AD), daily 600 mg benfotiamine significantly reduced the rise of blood AGE levels and slowed cognitive decline. ADAS-Cog scores increased 43% less in the benfotiamine group (trend, *p*≈0.13) and CDR-SB increased 77% less (*p* = 0.034) compared to placebo [190]. No drug-related adverse events occurred. These results suggest benfotiamine may slow AD-related decline and lower glycation [190]. A larger Phase II trial (18 months, 406 mild-AD patients, dose-ranging) is now underway (adaptive design, completion ~ 2027) [191]. Notably, a Chinese add-on trial in AD (*n* = 302 on donepezil) saw no overall benefit on ADAS-Cog, though a subgroup with moderate AD showed dose-dependent slowing of decline [192]. Furthermore, a medicinal chemistry study designed phenyl benzoxazole derivatives with dual AChE inhibitory and antioxidant activity. The most potent analog (compound 34) had an AChE IC₅₀ of 0.363 μM and significantly higher antioxidant effect than donepezil. In vivo and ex vivo assessments showed compound 34 markedly reversed scopolamine-induced memory deficits and oxidative stress at 5 mg/kg, effects comparable to donepezil [193].

Diabetes drugs (Metformin, GLP-1 agonists, insulin) are sometimes considered “indirect” anti-glycation therapies. A pilot 12-month RCT of metformin (1000 mg BID) in 80 overweight adults with amnestic MCI found memory improvement on one test (SRT total recall) favoring metformin (9.7 vs. 5.3 point gain, *p* = 0.02) [194], though other measures were unchanged. About 10–15% of subjects could not tolerate full dose [194]. Larger metformin trials are in progress (e.g., the MAP study, NCT04098666) to test its preventive effects on cognition. Similarly, GLP-1 receptor agonists (e.g., liraglutide, semaglutide) and intranasal insulin are being tested in AD (due to their metabolic/anti-inflammatory effects) though results are pending. These agents improve glucose metabolism and could reduce glycation, but they are not specifically AGE-targeting.

Several other anti-glycation agents like AGE breakers and carbonyl scavengers (e.g., aminoguanidine, pyridoxamine, alagebrium) have shown benefit in animal models of AD, but no large AD trials in humans have been completed. Carnosine supplementation (a natural glycation scavenger) improved cognition in some small studies of elderly subjects, but its role in AD has not been established. Overall, to date, no anti-glycation therapy has proven efficacy in AD. Completed trials (azeliragon, benfotiamine, small metformin pilot) have yielded mixed or null results. Trials in progress (high-dose benfotiamine, larger metformin studies, GLP-1 trials) will clarify if targeting metabolism/glycation can slow AD.

## Concluding Remarks

Glycation is a natural process that modifies protein structure and function as they age, contributing to the pathogenesis of neurodegenerative diseases. In AD, glycation exacerbates the toxicity of the Aβ peptide, a key component of amyloid plaques, leading to increased cognitive decline and neuronal damage.

Diabetes, characterized by elevated blood sugar levels, accelerates glycation, resulting in a higher accumulation of altered proteins and worsening neurodegenerative diseases like AD. This connection underscores the significant impact of metabolic disorders on brain health.

Targeting glycation processes presents a promising strategy for developing therapies to protect against neurodegenerative conditions, particularly in T2DM patients where increased glycation exacerbates the accumulation of AGEs and neurotoxicity. Anti-diabetic medications that influence glycation pathways could potentially facilitate the simultaneous management of both diabetes and AD.

Ongoing research into protein glycation in AD may lead to identifying new diagnostic biomarkers and treatment strategies, enhancing the ability to detect and manage AD more effectively.

By bridging glycation research with clinical practice, we predict there is great potential for developing interventions that improve outcomes for AD patients, enhance their quality of life, and possibly slow or prevent other neurodegenerative conditions associated with aging and diabetes.

## Data Availability

No datasets were generated or analysed during the current study.

## Abbreviations

AD: Alzheimer’s disease
Aβ: Amyloid beta
T2DM: Type 2 diabetes mellitus
AGE: Amyloid glycation end-product
NFT: Neurofibrillary tangles
APP: Amyloid precursor proteins
COX: Cyclooxygenase
LPS: Lipopolysaccharides
CML: Carboxymethyl lysine
CEL: Carboxyethyl lysine
SWATH-MS: Sequential Window Acquisition of All Theoretical Fragment Ion Spectra
AEP: Asparagine endopeptidase
RAGE: Receptor for advanced glycation end products
GSK-3: Glycogen synthase kinase-3
ROS: Reactive oxygen species
sRAGE: Soluble receptor for advanced glycation end products
UPS: Ubiquitin-proteasome system
AKRs: Aldo-keto reductases
FN3 K: Fructosamine-3-kinase
BMI: Body mass index
TNF-α: Tumor necrosis factor-alpha
MG: Methylglyoxal
AMPK: AMP-activated protein kinases
JNK: C-Jun N-terminal kinase pathway
IRS-1: Insulin receptor substrate-1
GSK-3β: Glycogen synthase kinase 3 beta
SCFA: Short chain fatty acid
MCI: Mild cognitive impairment
CSF: Cerebrospinal fluid
GLP-1: Glucagon-like peptide-1
GLP-1R: Glucagon-Like peptide-1 receptor
